# Proportions of interferon-γ-producing ascites lymphocytes in response to mycobacterial antigens: A help for early diagnosis of peritoneal tuberculosis in a low TB incidence country

**DOI:** 10.1371/journal.pone.0214333

**Published:** 2019-04-04

**Authors:** Sophie Henrard, Véronique Corbière, Liliane Schandené, Martine Ducarme, Anne Van Praet, Emmanuelle Petit, Mahavir Singh, Camille Locht, Violette Dirix, Françoise Mascart

**Affiliations:** 1 Immunodeficiencies Treatment Unit, Hôpital Erasme, Université Libre de Bruxelles (U.L.B.), Brussels, Belgium; 2 Laboratory of Vaccinology and Mucosal Immunity, Université Libre de Bruxelles (U.L.B.), Brussels, Belgium; 3 Immunobiology Clinic, Hôpital Erasme, Université Libre de Bruxelles (U.L.B.), Belgium; 4 INSERM, U1019, Lille, France; 5 CNRS, UMR8204, Lille, France; 6 Université de Lille, Lille, France; 7 Institut Pasteur de Lille, Centre d’Infection et d’Immunité de Lille, Lille, France; 8 Lionex Diagnostics and Therapeutics, Braunschweig, Germany; University of Cape Town, SOUTH AFRICA

## Abstract

**Background:**

Peritoneal tuberculosis (TB) remains difficult to diagnose because of its non-specific clinical features and the lack of efficient microbiological tests. As delayed diagnosis is associated with high mortality rates, new diagnostic tools are needed.

**Methods and findings:**

We investigated for 24 patients prospectively enrolled with a possible diagnosis of peritoneal TB, the diagnostic value of the analysis of IFN-γ production by peritoneal fluid lymphocytes in response to a short *in vitro* stimulation with mycobacterial antigens. The patients were classified in two groups: non-TB and confirmed or highly probable TB. Diagnosis of TB was based on microbiological and histopathological criteria and/or a favorable response to anti-TB treatment. The IFN-γ production by peritoneal CD4^+^ T lymphocytes was analyzed by flow cytometry after an overnight *in vitro* stimulation with three different mycobacterial antigens, purified protein derivative (PPD), heparin-binding haemagglutinin (HBHA) or early-secreted-antigen-target-6 (ESAT-6). The percentages of PPD-, HBHA- or ESAT-6-induced IFN-γ-producing peritoneal fluid CD4^+^ T lymphocytes were higher in the TB group than in the non-TB group (p = 0.0007, p = 0.0004, and p = 0.0002 respectively). Based on cut-off values determined by ROC curve analysis of the results from TB and highly probable TB compared to those of non-TB patients, the sensitivity of these three tests was 100% with a specificity of 92%.

**Conclusions:**

The analysis of mycobacterial-induced IFN-γ production by peritoneal lymphocytes is a promising tool to reliably and rapidly diagnose peritoneal TB. Further studies should be performed on larger cohorts of patients in high-TB-incidence countries to confirm the clinical value of this new diagnostic approach for peritoneal TB.

## Introduction

Tuberculosis (TB) is one of the world’s most lethal infectious diseases and peritoneal TB resulting from the growth of *Mycobacterium tuberculosis* complex bacteria in the peritoneum, is the sixth most frequent site of extra-pulmonary involvement. Peritoneal TB represents 1% to 2% of all clinical manifestations of TB and 31% to 58% of the abdominal TB, and is associated with pulmonary TB in 3.5% of cases [1]. Complications of septicemia, acute intestinal occlusion, infertility in women, are frequent [1], and its mortality rate ranges from 15% to 31% representing thus a significant public health problem, especially in endemic areas [2,3].

Peritoneal TB most commonly arises following the reactivation of latent *M*. *tuberculosis* infection from bacterial foci that have resided within the peritoneum after a hematogenous spread of *M*. *tuberculosis*, either from a primary lung focus or during miliary TB. Much less frequently, the organisms enter the peritoneal cavity after transmural migration from the infected small intestine or contiguously from TB salpingitis, and ascites develops secondary to the exudation of proteinaceous fluid from peritoneal tubercles [2,3]. Peritoneal TB is a subacute disease with unspecific symptoms evolving over a period of several weeks to months, so that the clinical presentation is rather insidious [1–3]. The most frequent clinical signs are ascites, abdominal pain, night sweats, fever and abdominal distension. TB peritonitis frequently occurs in patients with severe underlying medical conditions, such as end‐stage renal or liver disease, further adding to the diagnostic difficulty [2,3]. Laboratory blood tests, such as a moderate inflammatory syndrome and elevated carbohydrate antigen-125 concentrations, provide unspecific results. High ascites protein concentrations and elevated cellularity with a predominance of lymphocytes are informative but also unspecific. These laboratory results may easily be mis-interpreted for a malignant etiology [1–3]. The gold-standard for the diagnosis of peritoneal TB is a positive culture of mycobacteria from peritoneal fluids, but the sensitivity of this method is only 35% [2], so that diagnosis of peritoneal TB remains a real challenge. More sensitive diagnosis depends on a highly invasive procedure, which is the peritoneal biopsy performed by laparoscopy providing a positive culture in 92% to 98% of cases [3]. However, this procedure is associated with a significant risk for the patient, is costly and not available in resource-poor countries. Yet, early diagnosis is important, as delayed initiation of anti-TB therapy is associated with high mortality [2, 4].

A rapid, non-invasive, sensitive diagnostic test is urgently needed to guide decisions about the need for invasive laparoscopic examination or the initiation of empirical anti-TB treatment. Different approaches have already been evaluated, including various immunological tests. Among them, the most widely used remains the tuberculin skin test (TST), followed by the interferon-γ release assay (IGRA) performed on blood samples. However, these tests are not intended for the diagnosis of active TB for which they have a poor specificity, as they cannot distinguish active from latent TB and are not sensitive enough to diagnose peritoneal TB [5,6]. Another approach is based on the determination of adenosine deaminase (ADA) activity in ascites, that although not specific for TB, was reported to provide a high diagnostic accuracy of peritoneal TB [7]. More recently, new diagnostic approaches to diagnose extra-pulmonary TB have been described, based on the production of IFN-γ by lymphocytes collected from the site of infection. This approach is promising for pleural TB but also for pulmonary TB, as it provides a high degree of TB suspicion within 24 hours [8–10]. We report here on the diagnostic accuracy for rapid diagnosis of peritoneal TB of the percentages of IFN-γ-containing-CD4^+^ T lymphocytes after a short *in vitro* stimulation with purified protein derivative (PPD) or two different purified mycobacterial antigens, the heparin-binding haemagglutinin (HBHA) [11] and the early-secreted antigen target-6 (ESAT-6) [12].

## Material and methods

### Ethics statement

This study was approved by the ethics committee ULB-Hôpital Erasme (P2007/115, P2011/113 and P2016/252) and all the participants signed an informed consent form.

### Patients

Twenty-four patients living in Belgium, a low TB incidence country, hospitalized for fever and/or abdominal pain and with demonstrated ascites by abdominal ultrasound or computerized tomography scanning, were prospectively included in the study between 2010 and 2017. One patient (n°6) had also a bilateral pleural effusion. As all the patients were suspected to present active TB, peritoneal fluid was collected and sent to the laboratory as part of the classical diagnostic procedure, and the remaining fluid was tested for IFN-γ production by peritoneal CD4^+^ T lymphocytes in response to mycobacterial antigens.

### Mycobacterial induction of IFN-γ production

Peritoneal fluids were filtered and centrifuged as described for other fluids and red blood cells were lysed when necessary [10]. The cell recovery from peritoneal fluids was 2.2.10^6^ cells/ml for patients suffering from TB (median; 25^th^ - 75^th^ inter-quartiles: 1.5. 10^6^–4.6. 10^6^), and 0.019.10^6^ mononuclear cells /ml for non-TB patients (median; 25^th^ - 75^th^ inter-quartiles: 0.002. 10^6^–0.096. 10^6^). The peritoneal cell recovery was thus significantly different between the 2 groups of patients (p = 0.0047). The cell viability assessed by Trypan blue coloration was always > 90% and we considered that a sample cannot be further processed in case of cell mortality >50%. Five samples were discarded, as the number of lymphocytes was too small to perform the tests (less than 3.10^6^). The lymphocytes (2x10^6^ cells/ml, 500μl/condition) were incubated overnight with 4 μg/ml PPD (Staten Serum Institute, Copenhagen, Denmark), 10 μg/ml native HBHA purified as described (13)[13], 10 μg/ml ESAT-6 (Lionex Diagnostics & Therapeutics GmbH, Braunschweig, Germany), 0.5 μg/ml staphylococcal enterotoxin B (SEB, Sigma-Aldrich, Bornem, Belgium) or left unstimulated, as described [10]. Brefeldin A (10 μg/ml, Sigma-Aldrich) was added during the last 4 hours of incubation. After incubation and washing as described [10] the cells were stained with anti-CD3, anti-CD4 and anti- CD8 monoclonal antibodies (BD Biosciences, Erembodegem, Belgium) for 30 min. at 4°C, fixed and permeabilized using Caltag Fix and Perm reagent (ThermoFisher Scientific, Waltham, MA, USA) according to the manufacturer’s instructions. The cells were then labeled with an anti-IFN-γ PE antibody (BD Biosciences) for 30 min. at room temperature and acquired on a Navios flow cytometer (Beckman Coulter). The data were analyzed using the Kaluza software 1.5a (Beckman Coulter). The lymphocytes were gated based on the forward vs side scatter parameters (FSC, SSC), the dead cells and cell doublets were excluded before gating on the various cell subsets of interest, and a minimum of 100.000 CD4^+^ T lymphocytes was acquired. When blood was available (6 patients with TB and 8 classified as non-TB), an IFN-γ release assay was performed on peripheral blood mononuclear cells (PBMC) *in vitro* stimulated during 24 hrs with ESAT-6 as previously described [14].

### Statistical analysis

Data were analyzed with GraphPad Prism software, version 7.03 for Windows (GraphPad Software, La Jolla, CA, www.graphpad.com). The significance of the differences between two groups was determined using the non-parametric Mann-Whitney U test or the Wilcoxon test when paired values were compared, and a value of *P* < 0.05 was considered to be significant. Receiver Operating Characteristic (ROC) curves were established for each antigen by comparing results obtained for the patients with a confirmed or highly suspected diagnosis of TB to those from the patients with an alternative diagnosis, and cut-off values were determined to obtain optimal sensitivity and specificity. The significance of correlations was analyzed by the non- parametric Spearman test.

## Results

### Patient’s diagnosis based on classical criteria

The main demographic and clinical data from the individuals included in the final analysis (n = 19) and the main laboratory results are reported in Tables 1 and 2, respectively. The BCG vaccination status of the patients was mostly unknown, and the patients did not received antibiotics prior to sample collection, except patient n°16 who received amoxicilline/clavulanate. The classical laboratory analysis of all peritoneal fluids suggested a possible diagnosis of TB (high protein concentrations and mostly high percentages of lymphocytes). However, microscopic examination on a single smear was negative for *M*. *tuberculosis* for all ascites samples, and the culture was positive for *M*. *tuberculosis* only for one patient (n° 3). For two other patients, the culture was positive on the peritoneal biopsy, one for *M*. *tuberculosis*, and the other one for *M*. *bovis*, so that the diagnosis of peritoneal TB was microbiologically confirmed only for 3 patients (n° 1 to 3). Four other patients were treated for TB based on high clinical suspicion (n°4 to 7), and, as they all had a favorable clinical evolution with disappearance of the ascites, they were classified as highly probable TB patients. Both TB patients and highly probable TB patients were however grouped for further analysis. The other 12 patients were finally considered as non-TB cases, as they all had alternative diagnosis compatible with ascites (n° 8 to 19).

**Table 1:** Demographic and clinical characteristics of included patients.

Patient’s number	Gender	Age (years)	Ethnical origin	*Mtb* risk factors	Main symptoms	TST (mm)	Final diagnosis
1	M	35	Sub-Saharan	0	Fever Abdominal pain	17	TB
2	M	38	North African	0	Diarrhea Abdominal pain	ND	TB
3	M	27	Sub-Saharan	0	Diarrhea Abdominal pain	40	TB
4	M	31	Sub-Saharan	0	Abdominal pain	ND	TB
5	F	42	North African	Travel*	Abdominal pain	10	TB
6	F	14	Sub-Saharan	Travel**	Chest pain Dyspnea Abdominal pain	30	TB
7	M	36	North African	0	Weight loss Abdominal pain	20	TB
8	M	26	North African	Past TB	Weight loss Abdominal pain	ND	Gastric linitis Peritoneal carcinomatosis
9	M	60	Caucasian	0	Abdominal dullness	ND	Child C cirrhosis
10	F	72	Sub-Saharan	0	Weight loss Fever	ND	Myeloma
11	M	77	Caucasian	0	Abdominal pain	ND	Hepatocarcinoma
12	M	42	Caucasian	IV drug user	Abdominal pain	ND	Child C cirrhosis
13	F	70	Caucasian	0	Abdominal pain	ND	Peritoneal carcinomatosis
14	F	67	Caucasian	0	Abdominal pain	ND	Child C cirrhosis
15	M	45	North African	IV drug user	Abdominal dullness	ND	Hepatocarcinoma
16	F	63	Caucasian	0	Abdominal pain	ND	Peritoneal carcinomatosis
17	F	46	North African	0	Abdominal pain	ND	Metastatic breast cancer
18	M	59	Caucasian	0	Abdominal pain	ND	Hepatocarcinoma
19	M	65	Caucasian	HIV-infected	Abdominal dullness	ND	Hepatocarcinoma

M: male; F: female; TST: tuberculin skin test; TB: tuberculosis; IV: intravenous; ND: not done

*Recent travel to Marocco

**Recent arrival in Belgium (<12 months)

**Table 2:** Main biological results.

	Blood		Peritoneal fluid				Peritoneal biopsy		
Patient’s Number	CRP Conc. (N<10mg/L)	QFT Ag TB-Nil (N<0.35IU/ml)	Prot. Conc. (N<30g/L)	Lymphos (%)	*Mtb* Smear	*Mtb* Cult.	*Mtb* Smear	PCR	*Mtb* Cult.
1	7.2	1.12	60	32	-	-	-	-	+
2	11.0	ND*	60	73	-	-	-	-	+ *M*. *bovis*
3	29.0	1.77	60	51	-	+	ND	ND	ND
4	25.0	2.66	58	7	-	-	ND	ND	ND
5	62.0	ND**	48	ND	-	-	ND	ND	ND
6	53.0	0.11	67	54	-	-	ND	ND	ND
7	29.0	3.32	68	61	-	-	ND	ND	ND
8	53.0	1.08	45	45	-	-	ND	ND	ND
9	38.0	ND	23	6	-	-	ND	ND	ND
10	14.0	ND*	4	31	-	-	ND	ND	ND
11	19.0	ND*	14	48	-	-	ND	ND	ND
12	16.0	0.00	4	81	-	-	ND	ND	ND
13	63.0	ND*	12	5	-	-	ND	ND	ND
14	45.0	ND	37	25	-	-	ND	ND	ND
15	41.0	0.88	33	23	-	-	ND	ND	ND
16	31.0	ND*	5	6	-	-	ND	ND	ND
17	33.0	ND	13	19	-	-	ND	ND	ND
18	16.0	0.00	38	65	-	-	ND	ND	ND
19	74.0	0.00	35	ND	-	-	ND	ND	ND

*As the QFT test was not routinely available at the beginning of the study, a home-made interferon-γ-release assay in response to ESAT-6 was performed for some patients as described [14] and was negative (*) or positive (**) for patient n°5 with 172 pg/ml interferon-γ.

CRP: C-reactive protein; N = Normal value; QFT: QuantiFERON TB Gold in-Tube; conc: concentration; lymphos: lymphocytes; PCR: polymerase chain reaction; *Mtb*: *Mycobacterium tuberculosis*; ND: not done

### PPD-, HBHA-, and ESAT-6-induced IFN-γ production by ascites T lymphocytes

**Fig 1:**
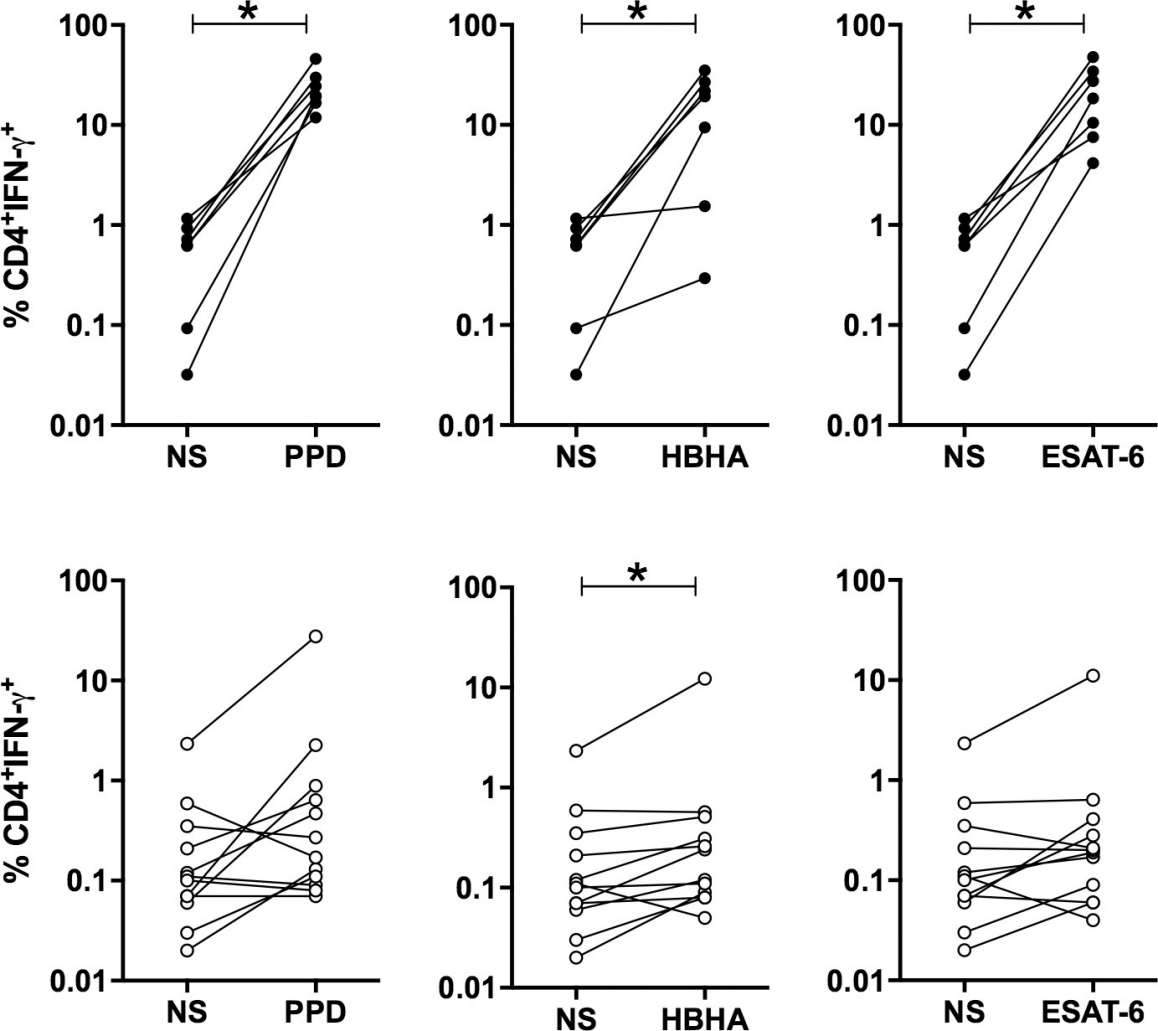
Percentages of IFN-γ^+^CD4^+^ T lymphocytes among non-stimulated (NS) compared to mycobacterial antigens-stimulated ascites lymphocytes. The percentages of IFN-γ-containing CD4^+^ lymphocytes were measured by flow cytometry after an overnight *in vitro* culture of the ascites lymphocytes left non-stimulated (NS) or stimulated with PPD (left panels), HBHA (middle panels) or ESAT-6 (right panels) for TB (upper panels) and non-TB patients (lower panels). Individual values obtained for each patient for lymphocytes left unstimulated and those stimulated with a mycobacterial antigen are linked. NS, non-stimulated. Results obtained in the presence or absence of antigens were compared by the Wilcoxon test *, *P*<0.05.

**Fig 2:**
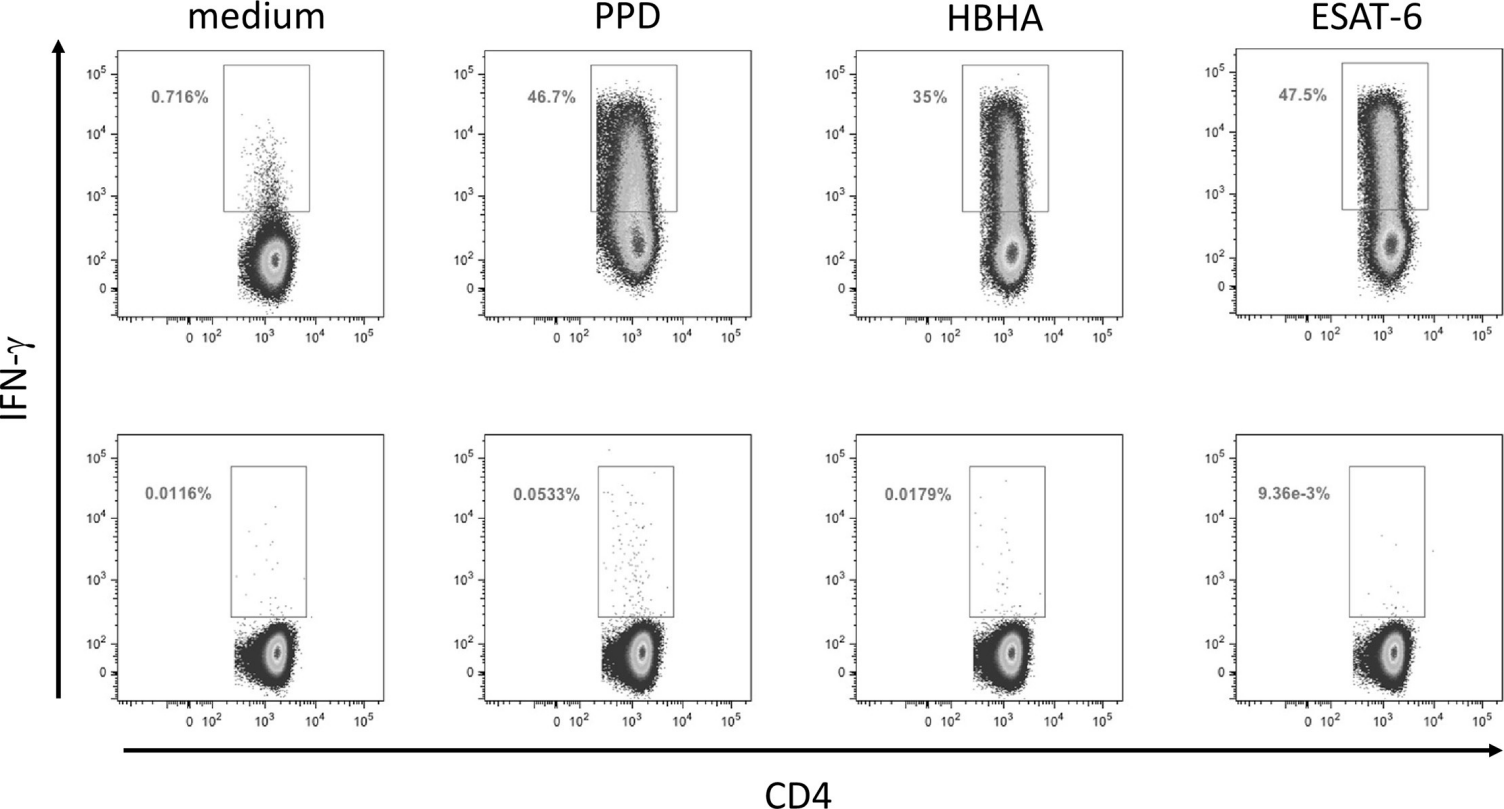
**Representative dot-plots of the flow cytometry analyses for one TB patient (upper panel) and one non-TB patient (lower panel).** Ascites lymphocytes were *in vitro* cultured overnight in the absence (medium) or the presence of PPD, HBHA or ESAT-6, as indicated. IFN-γ-producing CD4^+^T cells are shown after a sequential gating of Forward Scatter (FSC)/Side scatter (SSC), single cells and CD3^+^T lymphocytes. The percentages of IFN-γ^+^ T lymphocytes in each condition are indicated.

**Fig 3:**
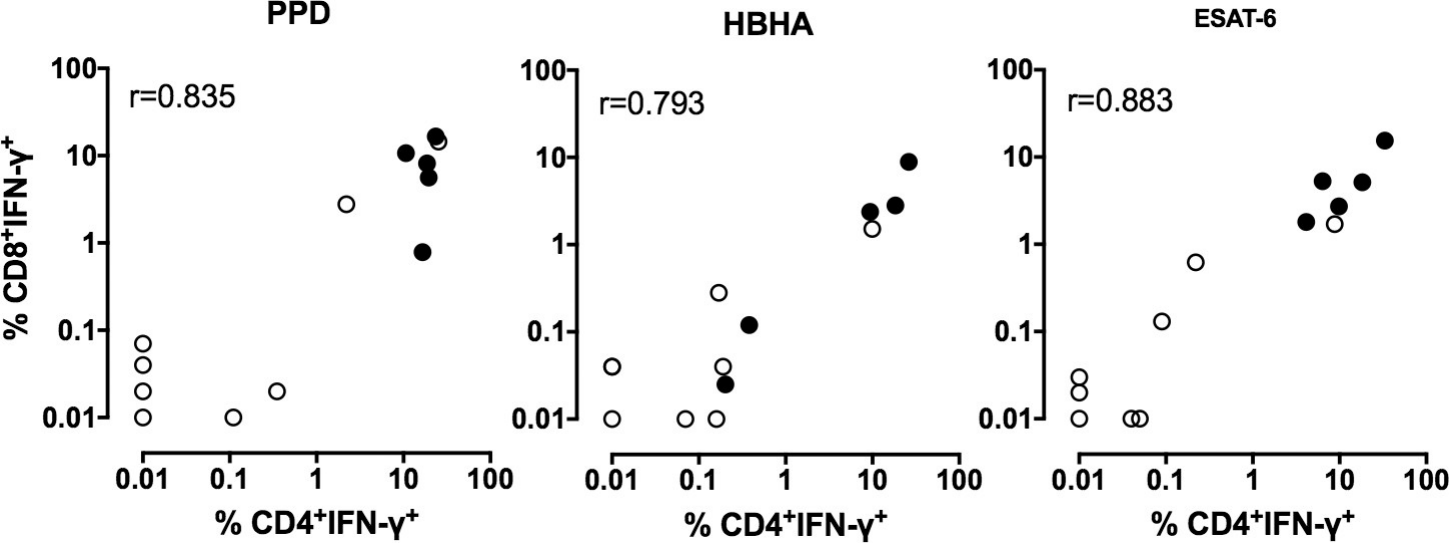
Correlations between the percentages of IFN-γ-containing CD4^+^ and CD8^+^ T lymphocytes induced by the mycobacterial antigens. Ascites lymphocytes were *in vitro* cultured overnight in the absence (medium) or the presence of PPD, HBHA or ESAT-6, and the percentages of IFN-γ-containing CD4^+^ and CD8^+^ T lymphocytes were analyzed by flow cytometry. The values shown are obtained by subtracting the values obtained in the absence of antigen stimulation from those obtained in response to mycobacterial antigens. Filled circles represent values from patients suffering from TB, whereas open circles represent values from non-TB patients. r: Spearman coefficient of correlation.

The peritoneal fluids from TB patients contained approximately 6 times more non-stimulated IFN-γ-producing CD4^+^ T lymphocytes than to those of non-TB patients (medians 0.630% and 0.105%, Fig 1, upper and lower panels, TB and non-TB respectively), whereas the proportion of IFN-γ-containing CD8^+^ T lymphocytes was similar in the two groups of patients (medians 0.09% and 0.08% for TB and non-TB). After *in vitro* incubation with PPD, HBHA or ESAT-6, the proportions of the IFN-γ-containing-CD4^+^ T lymphocytes from all TB patients were significantly enhanced compared to the non-stimulated cells (p<0.05, Figs 1 and 2, upper panels). In contrast, for the non-TB patients, a rise in the proportions of peritoneal IFN-γ-producing CD4^+^ T lymphocytes was only observed for some of them after *in vitro* stimulation with PPD, HBHA or ESAT-6 (Figs 1 and 2, lower panels). Among CD8^+^ T lymphocytes, there was a trend for higher proportions of PPD, HBHA, ESAT-6-induced IFN-γ-containing cells but the differences between stimulated and non-stimulated cells were not significant, neither for TB patients, nor for non-TB patients. The proportions of PPD, HBHA, ESAT-6-induced IFN-γ-containing CD8^+^ and CD4^+^ T lymphocytes were however correlated (Fig 3).

### Diagnostic potential of the measurement of IFN-γ-producing ascites lymphocyte proportions in response to mycobacterial antigens

**Fig 4:**
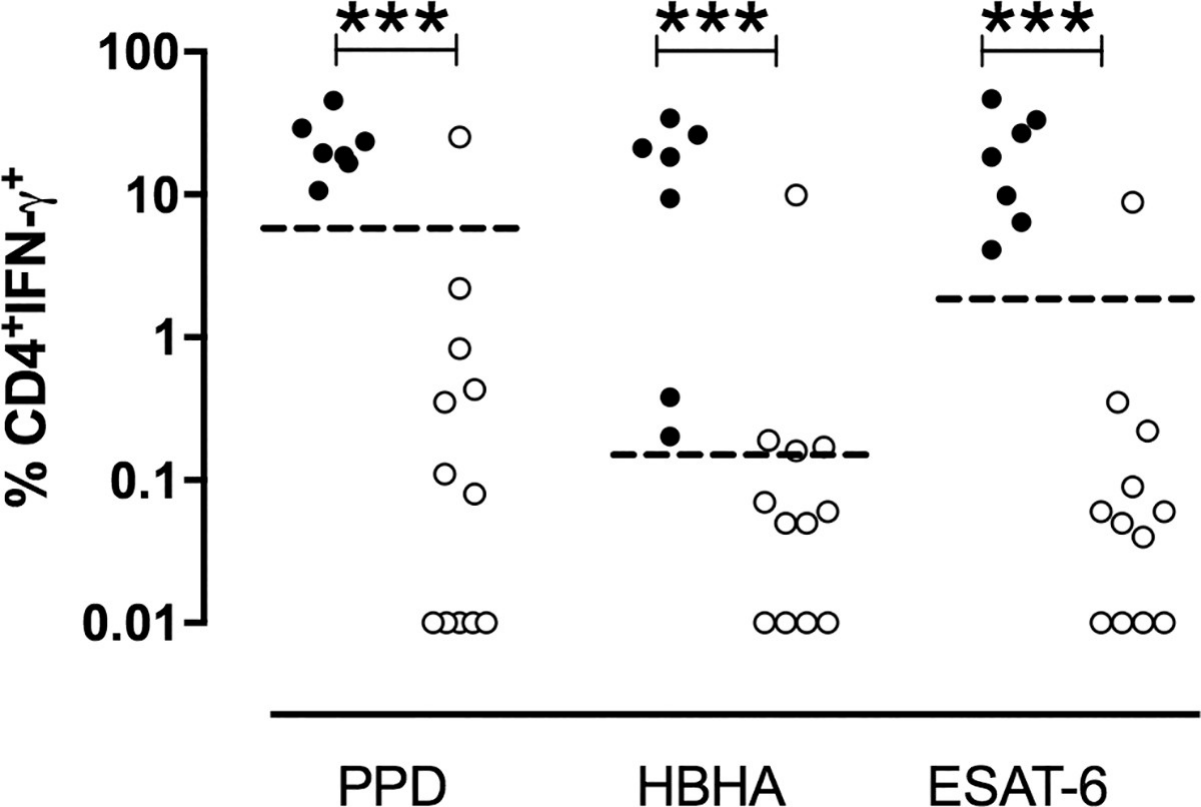
Percentages of IFN-γ-containing CD4^+^ ascites T lymphocytes induced by PPD, HBHA or ESAT-6. Ascites lymphocytes were *in vitro* cultured overnight in the absence or presence of PPD, HBHA ESAT-6, and the percentages of IFN-γ-containing CD4^+^ T lymphocytes were analyzed by flow cytometry. The values shown are obtained by subtracting the values obtained in the absence of antigen stimulation from those obtained in response to mycobacterial antigens. Filled circles represent values from patients suffering from TB, whereas open circles represent values from non-TB patients. Dotted horizontal lines represent the cut-offs determined by ROC analysis comparing the values for TB patients with those for non-TB patients. Values obtained for TB and non-TB patients were compared by Mann-Whitney U test. ***, *P*<0,001.

**Fig 5:**
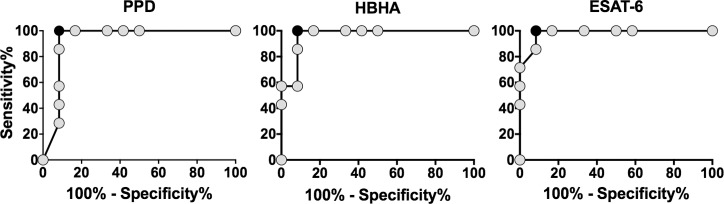
Receiver operator characteristics (ROC) curve analysis of the percentages of ascites IFN-γ-containing CD4^+^ lymphocytes obtained for TB and non-TB patients. The percentages of PPD-, HBHA-, and ESAT-6- induced IFN-γ-containing CD4^+^ lymphocytes were assessed by ROC analysis to define the most discriminant cut-off values between TB and non-TB patients. Cut-off values associated with optimal sensitivity and specificity were determined by the likelihood ratio analysis and are indicated by the black circles.

**Fig 6:**
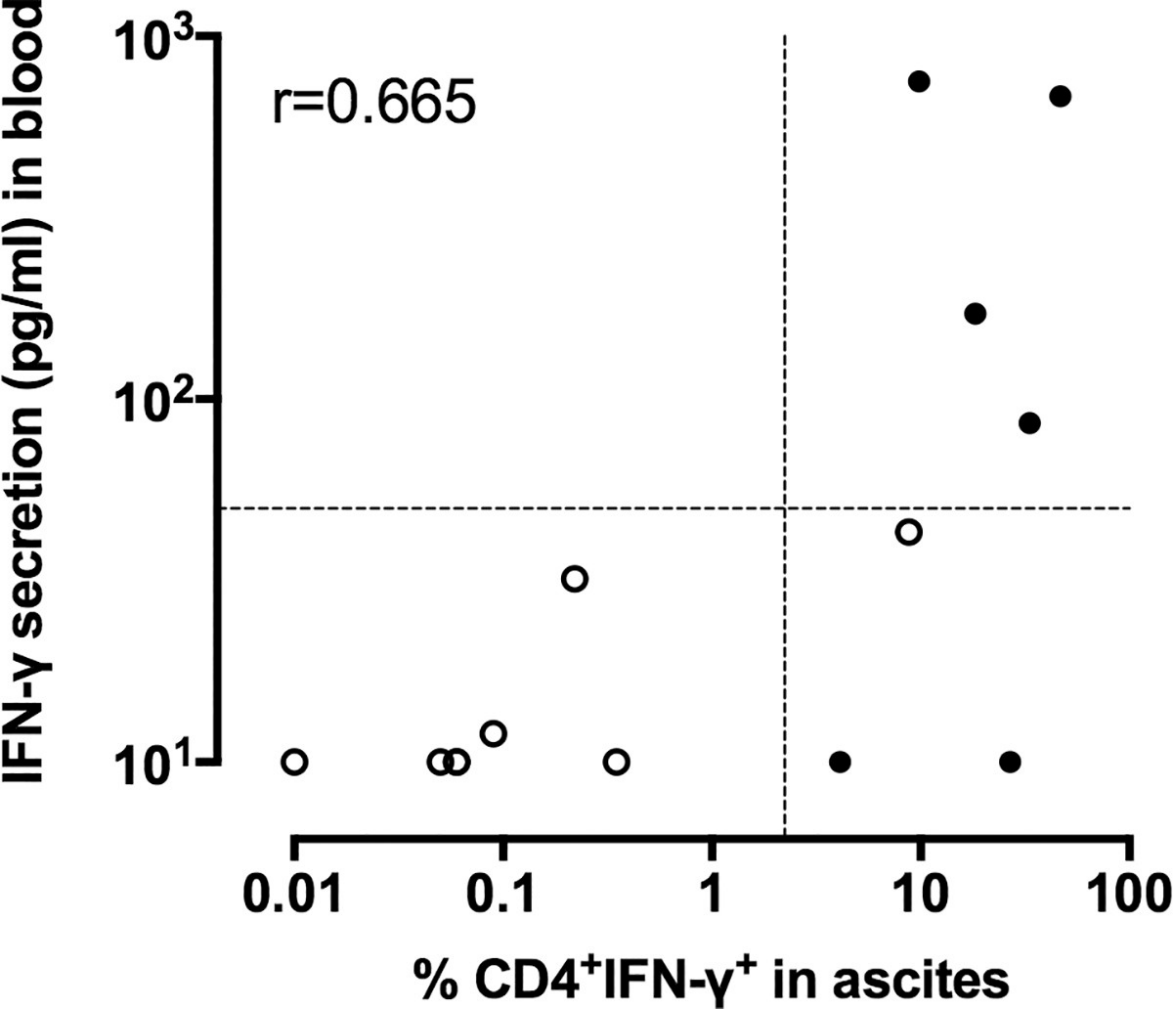
Comparison of the peripheral blood and peritoneal cell IFN-γ-responses to ESAT-6. Ascites lymphocytes were *in vitro* cultured overnight in the absence (medium) or the presence of ESAT-6, and the percentages of IFN-γ-containing CD4^+^ T lymphocytes were analyzed by flow cytometry. Peripheral blood mononuclear cells were *in vitro* cultured overnight in the absence or the presence of ESAT-6, and the concentration of IFN-γ-released was measured by ELISA. The values obtained in the absence of antigen stimulation were subtracted from those obtained in response to mycobacterial antigens for both the flow cytometry and the ELISA results. Filled circles represent values from patients suffering from TB, whereas open circles represent values from non-TB patients. r: Spearman coefficient of correlation.

TB patients had significantly higher proportions of IFN-γ-containing ascites CD4^+^ T lymphocytes after *in vitro* stimulation with mycobacterial antigens than non-TB patients (p = 0.0007 in response to PPD, p = 0.0003 in response to HBHA, and p = 0.0002 in response to ESAT-6, Fig 4). The percentages of cells shown in Fig 4 are those obtained after subtraction of the values obtained in the absence of antigen stimulation from those obtained in response to the mycobacterial antigens. The values obtained for the two groups of patients for each mycobacterial antigen were further compared by receiver operator characteristics (ROC) curve analysis to define the most discriminant cut-off values and evaluate the diagnostic potential of these tests for peritoneal TB (Fig 5). The areas under the curve (AUC) were of 0.94, 0.96, and 0.98 for PPD, HBHA, and ESAT-6, respectively. Optimal cut-off values providing 100% of sensitivity for the identification of patients with peritoneal TB or highly suspected peritoneal TB, were 6.44%, 0.20% and 2.23% of IFN-γ^+^ CD4^+^ T lymphocytes for PPD, HBHA and ESAT-6, respectively. They were all associated with a specificity of 92%. The values obtained in response to ESAT-6 provided the largest difference between TB and non-TB patients and, when blood was available, they were compared to the concentrations of IFN-γ released by PBMC from the same patients *in vitro* stimulated with ESAT-6. The results shown on Fig 6 indicated that only two thirds of the TB patients had a blood IFN-γ response to ESAT-6 above the cut-off value. In contrast, all but one patient classified as non-TB were negative for both the index case on ascites lymphocytes and for the blood IFN-γ release in response to ESAT-6. This patient (n°15) classified as non-TB showed in fact values on ascites lymphocytes above the cut-off for all three antigens. However, this patient was a diabetic intravenous drug user suffering from cirrhosis with an hepato-carcinoma and presenting with chronic renal disease, so that a peritoneal biopsy was contra-indicated. As both the blood ESAT-6-induced interferon-γ-release assay (Fig 6), and the QuantiFERON test performed on his blood (Table 2, patient n°15) were positive in addition to the positive results obtained on ascites lymphocytes, this suggests that this patient was infected with *M*. *tuberculosis* and may thus have been initially mis-classified by the clinician.

## Discussion

Peritoneal TB is associated with a high mortality rate and its diagnosis is often delayed due to unspecific clinical symptoms and low bacillary burden resulting often in negative acid-fast bacilli smear examination and negative culture [3]. In the present study conducted in a low TB incidence country, we report the diagnostic accuracy of the analysis of the IFN-γ production by peritoneal fluid CD4^+^ T lymphocytes in response to a short *in vitro* stimulation with mycobacterial antigens. Although in the absence of stimulation by mycobacterial antigens, TB patients had already higher proportions of IFN-γ-producing CD4^+^ ascites lymphocytes than the non-TB patients, this difference was not statistically significant and had therefore a low discriminatory power between TB and non-TB patients. CD8^+^ T lymphocytes also produced IFN-γ- in response to an *in vitro* stimulation with mycobacterial antigens, but the differences between the results obtained with or without antigenic stimulation were not significant with therefore low diagnostic relevance of these results.

IFN-γ production by fluid lymphocytes in response to an *in vitro* stimulation with mycobacterial antigens has already been suggested as a potential diagnostic aid for pulmonary TB, pleural TB and TB meningitis [8–10,15]. Two independent case reports also suggested a diagnostic potential of such tests for peritoneal TB [16, 17], whereas a large study performed in a high TB incidence country reported no added value of the T-SPOT.TB test (Oxford Immunotec) performed on ascites lymphocytes over the diagnostic accuracy provided by measuring the peritoneal fluid ADA [18]. However, only a sensitivity of 82% with a specificity of 79% was achieved by ADA measurements for the diagnosis of peritoneal TB in this study. Therefore, the authors proposed a two-step algorithm based on both T cell-based assays and ADA determinations to reach a correct classification of 67% of the patients [18]. ADA determinations are not available as a clinical setting in Belgium and we have chosen here to analyze the IFN-γ production by ascites lymphocytes using flow cytometry. This allowed us to focus on the CD4^+^ T lymphocytes, the major T lymphocyte subset producing IFN-γ in response to a short *in vitro* stimulation with mycobacterial antigens. By focusing on these cells, we observed extremely high proportions of CD4^+^ ascites T lymphocytes from TB patients producing IFN-γ in response to mycobacterial antigens, up to 47%. These extremely elevated responses may be due at least partially to the inhibition by ADA, known to be elevated in TB ascites, of CD4^+^ regulatory T cells that normally tend to inhibit extreme degrees of activation of CD4^+^ effector T lymphocytes [19]. Based on these results, we propose this new test as a valuable adjunct for the diagnosis of peritoneal TB. We have tested the inter-laboratory reproducibility of the assay by dividing some samples into two aliquotsand processing them in two independent laboratories for flow cytometry analysis. The percentages of CD4^+^ IFN-γ-containing T lymphocytes reported by the two laboratories were strongly correlated for all 3 antigens, with r values of 0.90, 0.71 and 0.81 for PPD, HBHA and ESAT-6, respectively (Spearman correlation test).

As results can be obtained within only 24 hours, and as flow cytometry is available in most clinical laboratories, this test provides very quickly useful data to guide decisions about the need for invasive laparoscopic examination and for the initiation of empirical anti-TB treatment. It should be particularly helpful for rapid differential diagnosis with confounding etiologies, such as ovarian cancer [20], and may also help to diagnose peritoneal TB sometimes associated with cancer [21]. This help cannot be obtained by blood tests like the QuantiFERON that can be negative in patients with peritoneal TB [6], as patient n°6 in this study, or other forms of TB [5], even with the QuantiFERON-TB Gold Plus [22]. On the contrary, the QuantiFERON test may be positive in subjects with latent TB presenting with ascites not considered as related to a *M*. *tuberculosis* infection (patients n° 8 and n°15), as this test cannot distinguish active from latent TB [5].

High risk populations for peritoneal TB include patients with AIDS or with other severe immunosuppressive conditions, including cirrhosis and continuous ambulatory peritoneal dialysis [23]. Diagnosis of peritoneal TB is further complicated for these patients, as peritoneal biopsy performed by laparoscopy may be contra-indicated, such as for patient n°15 of our study. The recent case report of a peritoneal TB being the cause of ascites in a patient with cirrhosis [24] lead us to consider that patient n°15 could in fact have been a miss-classification. If we consider patient n°15 as a patient with TB, this brings the specificity of the determination of the percentages of *M*. *tuberculosis* specific IFN-γ-containing ascites CD4^+^ T lymphocytes to 100% for the diagnosis of peritoneal TB.

The main limitation of this study is the relatively low number of patients included with a final diagnosis of peritoneal TB, a limitation inherent to the fact that the study was performed in a low TB incidence country. Moreover, only 3 cases of peritoneal TB were confirmed by positive microbiological results as, except in case of peritoneal biopsies obtained by laparoscopy and associated with a significant risk for the patient, only 35% of the peritoneal TB are confirmed by a positive *M*. *tuberculosis* culture on ascites [2]. However, the detailed clinical characterization of the patients allowed the clinicians to be confident in their classification of the patients as TB or not TB, and the good clinical and biological responses to anti-TB treatment confirmed that the patients classified as “highly probable TB” were indeed most likely suffering from a peritoneal TB. Only one patient may have been misclassified as non-TB as discussed above.

In conclusion, our study indicates that the analysis of the IFN-γ response of ascites CD4^+^ T lymphocytes to the mycobacterial antigens HBHA and ESAT-6 is a promising tool for rapid and reliable diagnosis of peritoneal TB, with a high degree of sensitivity and specificity. This diagnostic method may be very useful, especially in challenging situations, where pulmonary infection is absent. Further investigation with a larger cohort especially in a high TB-incidence country is warranted to confirm the results.
